# Genetically Predicted Levels of Circulating Inflammatory Cytokines and the Risk and Age at Onset of Parkinson’s Disease: A Two-Sample Mendelian Randomization Study

**DOI:** 10.3389/fnagi.2022.811059

**Published:** 2022-03-01

**Authors:** Yating Zhao, Xiaoqian Zhang, Na Guo, Dandan Tian, Chenguang Zhang, Changqing Mu, Chen Han, Ruixia Zhu, Jian Zhang, Xu Liu

**Affiliations:** ^1^Department of Neurology, First Affiliated Hospital of China Medical University, Shenyang, China; ^2^Key Laboratory of Cell Biology, Ministry of Public Health, Department of Cell Biology, China Medical University, Shenyang, China; ^3^Key Laboratory of Medical Cell Biology, Ministry of Education, China Medical University, Shenyang, China

**Keywords:** cytokines, inflammation, Mendelian randomization, macrophage inflammatory protein-1 beta, interleukin-16, Parkinson’s disease

## Abstract

Parkinson’s disease (PD) is widely considered to be a disabling neurodegenerative disorder, which has been ranked second worldwide just after Alzheimer’s disease. Until present, a wide range of studies has focused on the role of circulating inflammatory cytokines in the development of PD. However, the causal relationship between circulating inflammatory cytokines and the risk and age at the onset of PD has not been elucidated. Hence, to evaluate the effects of circulating inflammatory cytokines on the risk or age at the onset of PD more accurately, we conducted this two-sample Mendelian randomization (MR) study involving summary statistics from genome-wide association studies (GWASs). Totally, we included a GWAS for inflammatory cytokines (8,293 participants), a meta-analysis of GWASs for PD risk (482,730 participants), and a GWAS dataset for age at the onset of PD (17,996 patients with PD). A total of 149 and 131 polymorphisms for exploring relationships between 19 inflammatory cytokines and the risk and age at the onset of PD were obtained as instrumental variants. Then, we used a total of five MR methods, including inverse-variance weighted (IVW), Wald ratio, MR Egger regression, weighted median, and MR-pleiotropy residual sum and outlier (MR-PRESSO) methods. Finally, we found a causal association between circulating levels of macrophage inflammatory protein-1 beta (MIP1b) and PD risk in the IVW method (OR: 1.06; 95% CI: 1.02–1.10; *P* = 0.001). Meanwhile, other MR estimates by weighted median and MR-PRESSO methods yielded similar effect estimates. Besides, we identified a suggestive association of interleukin-16 (IL-16) levels with PD risk (OR: 1.08; 95% CI: 1.00–1.17; *P* = 0.037). For age at PD onset, there was no evidence supporting its correlation with inflammatory cytokines. Our findings implied that MIP1b and IL-16 may be novel biomarkers and promising therapeutic targets for PD development.

## Introduction

Parkinson’s disease (PD) is considered to be an aging-related neurodegenerative disease, characterized by a broad spectrum of clinical features, including tremor, bradykinesia, rigidity, postural instability, and dysautonomia (Jankovic, 2008). During the past decades, the number of individuals suffering from PD increased from 2.5 million in 1990 to 6.1 million in 2016 globally, and it brought great distress and economic burden to society and families (GBD 2016 Parkinson’s Disease Collaborators, 2018). In the United States alone, the overall annual cost related to PD was estimated to be US$51.9 billion in 2017 and was projected to continually increase and eventually surpass US$79 billion by 2037 (Yang et al., 2020). Thus, huge efforts have been made for exploring possible biomarkers and clarifying the pathogenesis for PD development (Schapira and Jenner, 2011; Lotankar et al., 2017).

Presently, an increasing number of evidence supported the vital role of inflammation in the pathogenesis of PD (Marogianni et al., 2020; Kline et al., 2021). It has been reported that systematic inflammation may destroy the permeability of the blood-brain barrier (BBB), activate microglia, and trigger neuroinflammation, which ultimately led to the degeneration of dopaminergic neurons and PD occurrence (Villaran et al., 2010; Joshi and Singh, 2018). Thus, as an indispensable part of the pathophysiological process of inflammation, inflammatory cytokines are suggested to be involved in PD development. Previous observational studies have demonstrated that circulating levels of several inflammatory cytokines were higher in individuals with PD than in healthy controls. For example, Pochmann et al. (2018) indicated that higher levels of monocyte chemotactic protein-1 (MCP-1) were detected in patients with PD than those in the control group. In a meta-analysis involving 2,654 individuals, elevated concentrations of interleukin-6 (IL-6) and IL-1β were observed in patients with PD but not in healthy controls (Qin et al., 2016). Moreover, several genetic polymorphisms near genes encoding MCP-1 and IL-1β were also indicated to be related to an increased risk of PD (Wahner et al., 2007; Wang et al., 2019). Additionally, it has been reported that higher expression of tumor necrosis factor-α (TNF-α) was associated with earlier onset of PD (Lindenau et al., 2017). However, due to the confounding factors, reverse causation, and limited sample size in these observational studies, the relationships between inflammatory cytokines and the risk and age at the onset of PD may be misled.

Mendelian randomization (MR) is considered to be a robust method, which could overcome the limitations of observational studies mentioned earlier by using genetic variants as instrumental variables and large-scale data from genome-wide association studies (GWASs). Hence, in this study, we introduced the MR method to assess the causal relationship between inflammatory cytokines and the risk and age at the onset of PD.

## Materials and Methods

### Study Design

**FIGURE 1:**
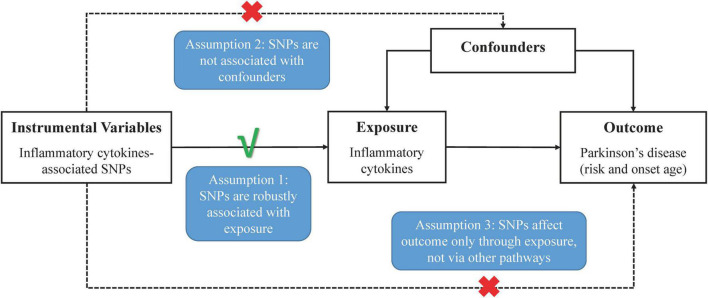
Schematic representation of two-sample Mendelian randomization analyses for circulating levels of inflammatory cytokines and risk of Parkinson’s disease.

In Figure 1, the overall design of this two-sample MR analysis is displayed. The study was based on publicly available data from GWASs on circulating inflammatory cytokines and PD, with detailed information listed in Supplementary Table 1. Since we used summary statistics from published studies, no additional ethical approval was required.

### Data Sources and Instrument Selection

Concerning PD risk, we included summary data from a GWAS meta-analysis involving IPDGC-NeuroX, UK Biobank, SGPD and IPDGC study with 33,674 cases and 449,056 controls (contributing studies outlined in Supplementary Table 1; Nalls et al., 2019). As for age at the onset of PD, estimates were extracted from a GWAS dataset including 17 cohorts with 17,996 cases with PD (Blauwendraat et al., 2019).

Regarding the instrument variables of inflammatory cytokines, we obtained genetic variants from one recently published GWAS on circulating inflammatory cytokines involving 8,293 European participants (Ahola-Olli et al., 2017). First, we obtained 6,368 single-nucleotide polymorphisms (SNPs) according to a genome-wide significance threshold of *P* < 5 × 10^–8^ and the false discovery rate (FDR) less than 5%. Then, for each cytokine, we pruned all selected SNPs in linkage disequilibrium (LD) (*r*^2^ < 0.1 in the European 1,000 genomes reference panel), retaining 199 genetic polymorphisms with the lowest *P*-value as an independent instrument. Next, to avoid pleiotropic effects, 38 SNPs associated with concentrations of more than one cytokine were excluded, leaving 161 polymorphisms focusing on 19 inflammatory cytokines for use. Finally, since 30 SNPs related to the circulating levels of cytokines were not available in the PD risk or age of PD onset datasets, we selected other SNPs in high LD for replacement in further MR analysis (*r*^2^ varying between 0.8 and 1). In the end, a total of 149 SNPs and 131 SNPs for detecting relationships between inflammatory cytokines and the risk and age at the onset of PD were included in this study separately. The detailed information of 19 cytokines and their related SNPs used as instrument variables is displayed in Supplementary Table 2.

### Statistical Analyses

First, for those cytokines with only one related SNP, the Wald ratio method was used to compute the MR estimates for the association between one-SD elevated circulating cytokine levels and PD risk or age of PD onset, respectively. When over one variant was included, the inverse-variance weighted (IVW) method, as principal analysis, was used to evaluate the potential causal relationships between circulating levels of inflammatory cytokines and the risk and age at the onset of PD. For further assessing the heterogeneity of the causal estimates across different SNPs, Cochran’s *Q*-test was applied in the IVW method. Subsequently, we evaluated the possibility that the overall MR estimate was driven by a single SNP using leave-one-out analysis (Noyce et al., 2019). Specifically, we reran the MR analysis by using all SNPs and excluding only one outlier SNP at each time. Furthermore, three supplementary analyses, including MR Egger regression, weighted median, and MR-pleiotropy residual sum and outlier (MR-PRESSO) approaches, were performed for investigating the presence of pleiotropy and further evaluating the causal relationships (Bowden et al., 2015, 2016; Verbanck et al., 2018). In addition, we calculated the *F*-statistics to quantify the strength of the selected instruments, all of which were above the threshold of the *F*-statistics (*F* > 10) typically recommended for MR analyses. As 19 exposures were involved, we set the statistically significant *P*-value threshold to 2.63 × 10^–3^ (0.05/19) after the Bonferroni correction. The suggestive association was identified as a *P*-value between the conventional significance level (0.05) and the Bonferroni-corrected significance level. All above statistical analyses were conducted by using the *R*-statistical software (version 4.0.3) with related *R* packages, including MR, two-sample MR, as well as MR-PRESSO.

## Results

### Circulating Inflammatory Cytokines and Parkinson’s Disease Risk

**FIGURE 2:**
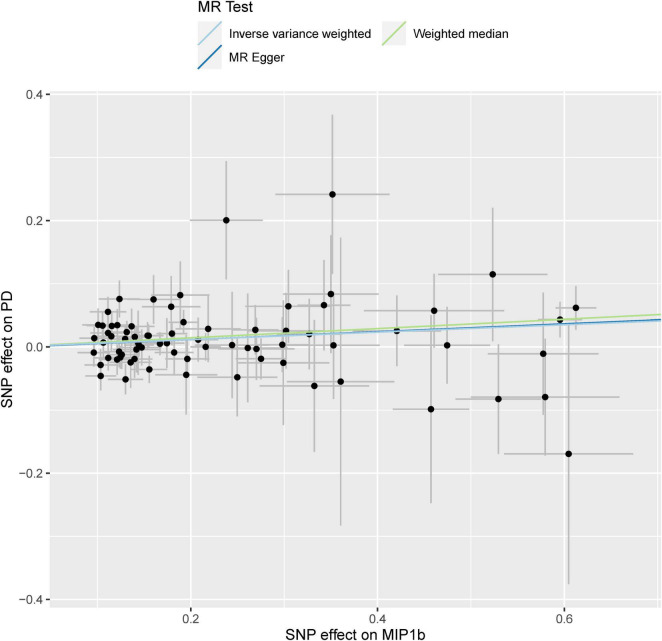
Scatterplot of genetic association with circulating levels of MIP1b against the genetic association with PD risk. Each black dot indicates an SNP, plotted by the estimate of SNP on the MIP1b levels and the estimate of SNP on PD risk with standard error bars. The slope of the line represents the causal relationship, and each method has a different line. PD, Parkinson’s disease; SNP, single-nucleotide polymorphism.

As displayed in Table 1 and Supplementary Figure 1, by using a significant threshold of 2.63 × 10^–3^ (Bonferroni correction for the correlation test of 19 cytokines), we found that genetically predicted one-SD increment in circulating MIP1b levels was associated with 6% higher risk of PD based on 74 SNPs in the IVW method (*OR*: 1.06; 95% *CI*: 1.02–1.10; *P* = 0.001). Furthermore, we did not observe any significant heterogeneity as measured by Cochran’s *Q*-test (*I*^2^ = 0.5%, *P* = 0.465). Subsequent leave-one-out analysis showed that no single SNP dominated the IVW point estimate (Supplementary Figure 2). Similarly, a significant association was found between circulating levels of MIP1b and PD risk in the MR-PRESSO method (*OR*: 1.06; 95% *CI*: 1.02–1.10; *P* = 0.002). Consistent with these results, suggestive evidence of an adverse effect of circulating MIP1b levels on PD risk was also observed by the weighted median method (*OR*: 1.08; 95% *CI*: 1.02–1.14; *P* = 0.014). Although the significant statistical association was not detected by MR Egger regression, the estimate was directionally consistent with other principal and supplementary analyses (Table 1 and Figure 2). Additionally, there was no evidence for potential pleiotropy according to the intercept assessed by MR Egger regression; meanwhile, no outlier SNP was detected using the MR-PRESSO method.

**TABLE 1:** MR analyses of genetically predicted levels of circulating inflammatory cytokines and risk of Parkinson’s disease.

Cytokines	No. of SNPs	OR (95% CI)	*P* for association	Heterogeneity test (*I*^2^, *P*)	MR-Egger (intercept, *P*)	*P* for MR-PRESSO global test
MIP1b						
Inverse variance weighted	74	1.06 (1.02–1.10)	0.001	0.5%, 0.465		
MR egger	74	1.06 (0.99–1.14)	0.088		–0.001, 0.908	
Weighted median	74	1.08 (1.02–1.14)	0.014			
MR-PRESSO (raw, 0 outliers)	74	1.06 (1.02–1.10)	0.002			0.491
TRAIL						
Inverse variance weighted	25	0.98 (0.91–1.05)	0.556	41.7%, 0.016		
MR egger	25	0.93 (0.84–1.03)	0.177		0.019, 0.200	
Weighted median	25	0.99 (0.91–1.07)	0.813			
MR-PRESSO (raw, 0 outliers)	25	0.98 (0.91–1.05)	0.562			0.016
IL18						
Inverse variance weighted	8	1.07 (0.97–1.19)	0.182	36.7%, 0.136		
MR egger	8	1.39 (1.07–1.79)	0.012		–0.061, 0.037	
Weighted median	8	1.10 (0.98–1.23)	0.103			
MR-PRESSO (raw, 0 outliers)	8	1.07 (0.97–1.19)	0.224			0.144
MCP1						
Inverse variance weighted	7	0.97 (0.86–1.10)	0.657	0.0%, 0.776		
MR egger	7	1.13 (0.86–1.49)	0.376		–0.022, 0.233	
Weighted median	7	1.01 (0.86–1.18)	0.923			
MR-PRESSO (raw, 0 outliers)	7	0.97 (0.89–1.06)	0.569			0.726
GROa						
Inverse variance weighted	6	0.98 (0.91–1.06)	0.634	0.0%, 0.455		
MR egger	6	1.01 (0.81–1.27)	0.898		–0.009, 0.755	
Weighted median	6	0.94 (0.85–1.04)	0.224			
MR-PRESSO (raw, 0 outliers)	6	0.98 (0.91–1.06)	0.644			0.451
Eotaxin						
Inverse variance weighted	5	0.94 (0.82–1.09)	0.441	0.0%, 0.805		
MR egger	5	0.94 (0.49–1.82)	0.855		0.001, 0.988	
Weighted median	5	1.01 (0.85–1.21)	0.894			
MR-PRESSO (raw, 0 outliers)	5	0.94 (0.86–1.04)	0.293			0.743
TNFb						
Inverse variance weighted	4	1.02 (0.95–1.10)	0.541	0.0%, 0.434		
MR egger	4	1.11 (0.79–1.56)	0.532		–0.088, 0.612	
Weighted median	4	1.03 (0.95–1.12)	0.430			
MR-PRESSO (raw, 0 outliers)	4	1.02 (0.95–1.10)	0.568			0.644
CTACK						
Inverse variance weighted	4	1.03 (0.92–1.15)	0.593	21.8%, 0.280		
MR egger	4	1.05 (0.77–1.44)	0.746		–0.007, 0.877	
Weighted median	4	1.05 (0.93–1.17)	0.444			
MR-PRESSO (raw, 0 outliers)	4	1.02 (0.92–1.15)	0.630			0.363
IL16						
Inverse variance weighted	3	1.08 (1.00–1.17)	0.037	0.0%, 0.705		
MR egger	3	1.06 (0.94–1.19)	0.339		0.010, 0.613	
Weighted median	3	1.07 (0.99–1.17)	0.095			
IL2ra						
Inverse variance weighted	3	1.03 (0.95–1.11)	0.538	0.0%, 0.574		
MR egger	3	0.91 (0.69–1.20)	0.506		0.062, 0.376	
Weighted median	3	1.03 (0.95–1.12)	0.511			
IP10						
Inverse variance weighted	2	0.92 (0.73–1.15)	0.448	0.0%, 0.934		
IFNg1						
Wald ratio	1	0.88 (0.56–1.40)	0.599			
IL10						
Wald ratio	1	0.87 (0.59–1.28)	0.474			
IL12p70						
Wald ratio	1	1.13 (0.77–1.65)	0.531			
IL17						
Wald ratio	1	0.99 (0.65–1.51)	0.979			
MCSF						
Wald ratio	1	0.83 (0.65–1.06)	0.127			
MIF						
Wald ratio	1	0.98 (0.79–1.23)	0.877			
MIG						
Wald ratio	1	1.04 (0.82–1.32)	0.754			
RANTES						
Wald ratio	1	0.95 (0.62–1.44)	0.803			

*SNP, single-nucleotide polymorphism; OR, odds ratio; CI, confidence interval; MR-PRESSO, Mendelian randomization pleiotropy residual sum and outlier.*

Regarding interleukin-16 (IL-16), we identified a suggestive association between circulating IL-16 levels and PD risk in IVW analysis. Specifically, for one SD increment of IL-16 levels, the OR of PD risk was 1.08 (95% *CI*: 1.00–1.17; *P* = 0.037). Further analysis showed a lack of evidence of heterogeneity among SNPs in the suggestive association of IL-16 with PD risk (*I*^2^ = 0, *P* = 0.705). Meanwhile, additional supplementary analysis displayed a similar causal trend with the estimation using the IVW method. In addition, no potential pleiotropy was detected using the MR Egger method (Table 1).

Apart from MIP1b and IL-16, the other 17 cytokines (TRAIL, MCP1, GROa, eotaxin, TNFb, CTACK, IL2ra, IP10, IFNg1, IL10, IL12p70, IL18, IL17, MCSF, MIF, MIG, and RANTES) were not shown to be associated with PD risk in the main IVW analysis and three supplementary analyses (Table 1). For each cytokine, no marked heterogeneity was found between related SNPs, except for TRAIL (*I*^2^ = 41.7%, *P* = 0.016). Meanwhile, the *P*-values for the intercepts from Egger regression did not demonstrate any pleiotropy, while IL-18 was the exception (*P* = 0.037). Further, the MR-PRESSO test showed that no outlier was found for all cytokines, except for TRAIL (*P* = 0.016).

### Circulating Inflammatory Cytokines and Age at the Onset of Parkinson’s Disease

Except for identifying the correlation between inflammatory cytokines and PD risk mentioned earlier, we also detected the causal relationships between the circulating levels of these cytokines and the age of PD onset. Unfortunately, there was no evidence to support the causal relationships between these 19 cytokines and age at the onset of PD (Table 2). Moreover, no statistical evidence of instrument heterogeneity was detected using Cochran’s *Q*-test except for IP10 (*I*^2^ = 79.1%, *P* = 0.029). When applying MR Egger regression, evidence of potential pleiotropy was only observed in GROa (*P* = 0.029), while other inflammatory cytokines did not show any pleiotropy. Besides, no SNP was detected as an outlier through the MR-PRESSO test (Table 2).

**TABLE 2:** MR analysis of genetically predicted levels of circulating inflammatory cytokines and age at onset of Parkinson’s disease.

Cytokines	No. of SNPs	Beta (95% CI)	*P* for association	Heterogeneity test (*I*^2^, *P*)	MR-egger (intercept, *P*)	*P* for MR-PRESSO global test
MIP1b						
Inverse variance weighted	65	–0.02 (–0.26–0.21)	0.854	0.0%, 0.516		
MR egger	65	0.00 (–0.49–0.48)	0.986		–0.004, 0.934	
Weighted median	65	–0.04 (–0.40–0.32)	0.840			
MR-PRESSO (raw, 0 outliers)	65	–0.02 (–0.26–0.21)	0.853			0.534
TRAIL						
Inverse variance weighted	21	–0.01 (–0.36–0.33)	0.938	0.0%, 0.939		
MR Egger	21	–0.26 (–0.77–0.26)	0.324		0.095, 0.208	
Weighted median	21	0.04 (–0.42–0.50)	0.861			
MR-PRESSO (raw, 0 outliers)	21	–0.01 (–0.27–0.25)	0.919			0.950
IL18						
Inverse variance weighted	7	–0.19 (–0.80–0.43)	0.553	20.5%, 0.273		
MR Egger	7	–0.44 (–2.43–1.54)	0.661		0.061, 0.787	
Weighted median	7	–0.29 (–0.98–0.40)	0.411			
MR-PRESSO (raw, 0 outliers)	7	–0.19 (–0.80–0.43)	0.575			0.328
MCP1						
Inverse variance weighted	7	–0.39 (–1.19–0.41)	0.342	0.0%, 0.917		
MR egger	7	0.33 (–1.56–2.22)	0.732		–0.106, 0.409	
Weighted median	7	–0.13 (–1.09–0.83)	0.796			
MR-PRESSO (raw, 0 outliers)	7	–0.39 (–0.86-0.08)	0.153			0.922
GROa						
Inverse variance weighted	6	–0.52 (–1.14–0.11)	0.104	33.9%, 0.182		
MR egger	6	0.90 (–0.47–2.28)	0.197		–0.397, 0.029	
Weighted median	6	–0.43 (–1.07–0.21)	0.184			
MR-PRESSO (raw, 0 outliers)	6	–0.52 (–1.14–0.11)	0.165			0.216
Eotaxin						
Inverse variance weighted	4	–0.10 (–1.12–0.91)	0.844	0.0%, 0.966		
MR egger	4	0.57 (–4.24–5.38)	0.816		–0.087, 0.779	
Weighted median	4	0.00 (–1.20–1.21)	0.995			
MR-PRESSO (raw, 0 outliers)	4	–0.10 (–0.40–0.20)	0.556			0.966
CTACK						
Inverse variance weighted	4	0.12 (–0.54–0.78)	0.715	11.5%, 0.335		
MR egger	4	0.67 (–1.08–2.42)	0.453		–0.165, 0.500	
Weighted median	4	0.25 (–0.51–1.01)	0.516			
MR-PRESSO (raw, 0 outliers)	4	0.12 (–0.54–0.78)	0.739			0.390
IL16						
Inverse variance weighted	3	0.41 (–0.11–0.93)	0.125	0.0%, 0.839		
MR egger	3	0.59 (–0.22–1.40)	0.151		–0.079, 0.558	
Weighted median	3	0.40 (–0.17–0.96)	0.166			
TNFb						
Inverse variance weighted	2	0.03 (–0.53–0.60)	0.905	0.0%, 0.902		
IL2ra						
Inverse variance weighted	2	0.45 (–0.33–1.22)	0.107	50.2%, 0.156		
IP10						
Inverse variance weighted	2	0.43 (–2.65–3.51)	0.547	79.1%, 0.029		
IFNg1						
Wald ratio	1	–0.79 (–4.24–2.67)	0.654			
IL10						
Wald ratio	1	–0.06 (–2.57–2.46)	0.965			
IL12p70						
Wald ratio	1	0.44 (–2.12–2.99)	0.738			
IL17						
Wald ratio	1	–1.18 (–3.80–1.45)	0.379			
MCSF						
Wald ratio	1	–0.02 (–1.56–1.52)	0.983			
MIF						
Wald ratio	1	–1.09 (–2.60–0.41)	0.153			
MIG						
Wald ratio	1	–0.53 (–2.12–1.06)	0.510			
RANTES						
Wald ratio	1	0.87 (–1.56–3.30)	0.484			

*SNP, single-nucleotide polymorphism; CI, confidence interval; MR-PRESSO, Mendelian randomization pleiotropy residual sum and outlier.*

## Discussion

Parkinson’s disease is a progressive and disabling neurodegenerative disease that mainly affects individuals in their later years of life and its course may vary from 6.9 to 14.3 years (Macleod et al., 2014; Marras et al., 2018). It has been reported that in 2016 alone, PD caused 211,296 deaths and 3.2 million disability-adjusted life-years globally (GBD 2016 Parkinson’s Disease Collaborators, 2018). Despite the long course of the disease and its huge impact on life expectance for elders, the intervention for PD prevention and therapy is still deficient, and the biological mechanism underlying PD etiology is not yet well understood. Thus, we took advantage of a two-sample MR analysis to investigate the causal role of circulating inflammatory cytokines in the risk and age at the onset of PD. In this study, higher MIP1b levels showed a causal relationship with the increased risk of PD, whereas another cytokine, IL-16, displayed a suggestive association.

Macrophage inflammatory protein-1 beta, also known as CCL4, is a chemotactic protein with 69 amino acids produced by various cells, including natural killer cells, T cells, B cells, and neutrophils (Menten et al., 2002; Mukaida et al., 2020). A previous study by Zhu et al. (2014) demonstrated that in the transgenic mice model of Alzheimer’s disease, MIP1b mRNA was 18 times higher than that of wild-type mice, indicating that MIP1b might be involved in the development of Alzheimer’s disease. In addition, MIP1b was also reported to be related to other neurodegenerative diseases, including multiple system atrophy and amyotrophic lateral sclerosis (Compta et al., 2019; Martinez et al., 2020). This evidence implied that MIP1b might participate in the initiation and progression of a neurodegenerative disorder. As for PD, in the past few decades, limited observational studies provided evidence of a possible association between MIP1b and PD risk. According to a previous case-control study involving 50 participants by Calvani et al. (2020), circulating levels of MIP1b were 2-fold higher in patients with PD than in controls, suggesting that it could be considered a possible biomarker for PD occurrence. Additionally, according to Brockmann’s study of 142 patients with PD, higher serum levels of MIP1b may be associated with more severe non-motor symptoms of PD, including cognitive impairment, sleep behavior disorder, and orthostatic dysfunction (Brockmann et al., 2017). Although these studies indicated an association between circulating levels of MIP1b and the occurrence and progression of PD, these results from observational studies were ambiguous and questionable. The reasons were as follows: due to the small sample size and potential confounding, the results of observational studies may be biased. Besides, the relationship between circulating levels of MIP1b and PD risk obtained from observational studies could not be determined as a causal association or even may even be a reverse causality. Therefore, we conducted this MR analysis and reached a reliable result that one-SD increment in the circulating levels of MIP1b may increase the risk of PD by 6%, indicating that lowering the levels of MIP1b may be a promising therapeutic strategy for PD.

From the perspective of biological mechanism, MIP1b may participate in the pathophysiology process of PD through the following pathways. On the one hand, MIP1b could induce the activation of macrophages by binding to its specific receptor, CCR5 (Cheung et al., 2009; Saika et al., 2012). Then, the activated macrophage might release high levels of pro-inflammatory cytokines, such as IL-1β, TNF-α, IL-6, and MIP-1α (Saika et al., 2012; Shapouri-Moghaddam et al., 2018). These cytokines could further disrupt the permeability of brain microvascular endothelial cells and the integrity of BBB (Al-Obaidi and Desa, 2018; Morris et al., 2018). On the other hand, MIP1b not only can immobilize glycosaminoglycans on the apical surface of microvascular endothelial cells but can also bind to the basal surfaces of endothelial cells as well as the subendothelial matrix. Both of the connections contributed to the establishment and maintenance of MIP1b gradients to help various circulating inflammatory cells pass through the BBB and enter the central nervous system (CNS) (Shukaliak and Dorovini-Zis, 2000). Subsequently, in response to these inflammatory mediators, microglia were activated and functionally polarized toward the pro-inflammatory M1 phenotype, which further induced the secretion of inflammatory cytokines, such as TNF-α, MCP-1, and IL-1β (Qin et al., 2007; Jo et al., 2017; Pajares et al., 2020). These chronically elevated inflammatory cytokines and activated inflammatory cells may, in turn, lead to chronic self-perpetuating neuroinflammation and eventually end up in the development of PD (Collins et al., 2012).

Regarding IL-16, it is a protein with 631 amino acids and is generally accepted to be a chemotactic cytokine for T cells (Cruikshank et al., 2000; Skundric et al., 2015). In our study, we found a suggestive association that one-SD increment in the circulating levels of IL-16 was associated with an 8% increase in PD risk. This could be explained by the following reasons: Similar to MIP1b, elevated IL-16 levels might also promote macrophages to release higher levels of pro-inflammatory cytokines and contribute to BBB breakdown (Persidsky et al., 2006; Huang et al., 2019). Besides, IL-16 could induce the activation of protein kinase C (PKC) and its translocation from cytosol to the membrane (Parada et al., 1996). Later, the PKC might phosphorylate the tight junction proteins of BBB leading to BBB damage (Li et al., 2018). Moreover, IL-16 could potentiate the inflammatory response by stimulating T cells to produce more pro-inflammatory cytokines (Skundric et al., 2015). Altogether, the production of pro-inflammatory cytokines, activation of immune cells, and continuous chronic inflammatory response could lead to PD occurrence.

There were several advantages involved in our study. This was the first MR analysis for clarifying the causality of inflammatory cytokines levels with the risk and age at the onset of PD. Another merit was the two-sample MR method that provided robust causal associations by minimizing confounding factors and avoiding reverse causality. Besides, we used data from publicly available GWASs, which included a large number of patients with PD and controls, thus affording a great power to explore the causal relationship. Finally, except for the IVW method, we also used three supplementary analyses, including MR Egger regression, weighted median, and MR-PRESSO approaches. Directional consistency of the results across multiple MR approaches reinforced the reliability of associations. Nevertheless, the results of this MR analysis should be noted in the context of the following limitations. First, since potential pleiotropy, outlier, and heterogeneity can be detected, the relationship between inflammatory cytokines, including TRAIL, IP10, IL-18, and GROa, and the risk or age at the onset of PD should be interpreted with caution. Second, we used summary data from GWASs of mostly European adults, which limited the generality of the observed causal associations to other populations with different genetic backgrounds. Third, cytokines are not something constitutively expressed in the body. During inflammation, specific inflammatory cytokines are induced to a high level but for only a short period. All of those dynamic changes cannot be addressed by MR analysis. Fourth, we focused on the causal effect of peripheral cytokines levels on the risk or age at the onset of PD and supported that systematic peripheral inflammation and chronic cytokine disturbance may contribute to the development of PD. However, a recent study by Wijeyekoon et al. (2020) suggested that some cytokines in the CSF did not correlate well with blood. Thus, our analysis cannot reveal the potential causal relationship between the levels of inflammatory cytokines in CSF and the risk or age at the onset of PD. Last but not the least, not all inflammatory cytokines were analyzed in our MR analysis due to the exclusion criteria and the limited number of cytokines analyzed in the previous GWAS.

## Conclusion

In conclusion, this MR analysis showed a causal relationship between circulating levels of MIP1b and PD risk and a suggestive association between IL-16 and PD. Additionally, there was no evidence in support of causality between inflammatory cytokines and age at the onset of PD. Our findings brought new insights into the pathogenesis of PD and indicated that MIP1b and IL-16 could be regarded as novel biomarkers and potential therapeutic targets for PD development.

## Data Availability Statement

The original contributions presented in the study are included in the article/Supplementary Material, further inquiries can be directed to the corresponding author/s.

## Author Contributions

XL designed the research. YZ and XZ had full access to all the data in the study and took responsibility for the integrity of the data and the accuracy of the data analysis. YZ and NG wrote the manuscript and performed the data analysis. All authors contributed to the statistical analysis, critically reviewed the manuscript during the writing process, and approved the final version to be published.

## Conflict of Interest

The authors declare that the research was conducted in the absence of any commercial or financial relationships that could be construed as a potential conflict of interest.

## Publisher’s Note

All claims expressed in this article are solely those of the authors and do not necessarily represent those of their affiliated organizations, or those of the publisher, the editors and the reviewers. Any product that may be evaluated in this article, or claim that may be made by its manufacturer, is not guaranteed or endorsed by the publisher.

## Supplementary Material

Supplementary Figure 1 Forest plot of the causal effect estimates of MIP1b-associated SNPs on PD risk. Each black dot indicates the causal estimate of MIP1b on PD using an SNP by the Wald ratio method with 95% confidence interval bars. The MR estimate using all SNPs with the IVW method is also shown (red dot with 95% confidence interval bars). PD, Parkinson’s disease; SNP, single-nucleotide polymorphism; MR, Mendelian randomization; IVW, inverse-variance weighted.

Supplementary Figure 2 Leave-one-out analysis of MIP1b-associated SNPs on PD risk. It was conducted to determine whether the causal effect of circulating MIP1b levels on PD risk was disproportionately influenced by a single SNP. The black dot and bars indicate the MR estimate and 95% confidence interval when the specific SNP is removed. The overall MR analysis, including all SNPs, is also presented for comparison (red dot with 95% confidence interval bars). SNP, single-nucleotide polymorphism; PD, Parkinson’s disease; MR, Mendelian randomization.
