# Supplementary figures and images for: Genetically Predicted Levels of Circulating Inflammatory Cytokines and the Risk and Age at Onset of Parkinson’s Disease: A Two-Sample Mendelian Randomization Study
Source: Front Aging Neurosci. 2022 Mar 1;14:811059. doi: 10.3389/fnagi.2022.811059 (PMC8923644; doi:10.3389/fnagi.2022.811059)

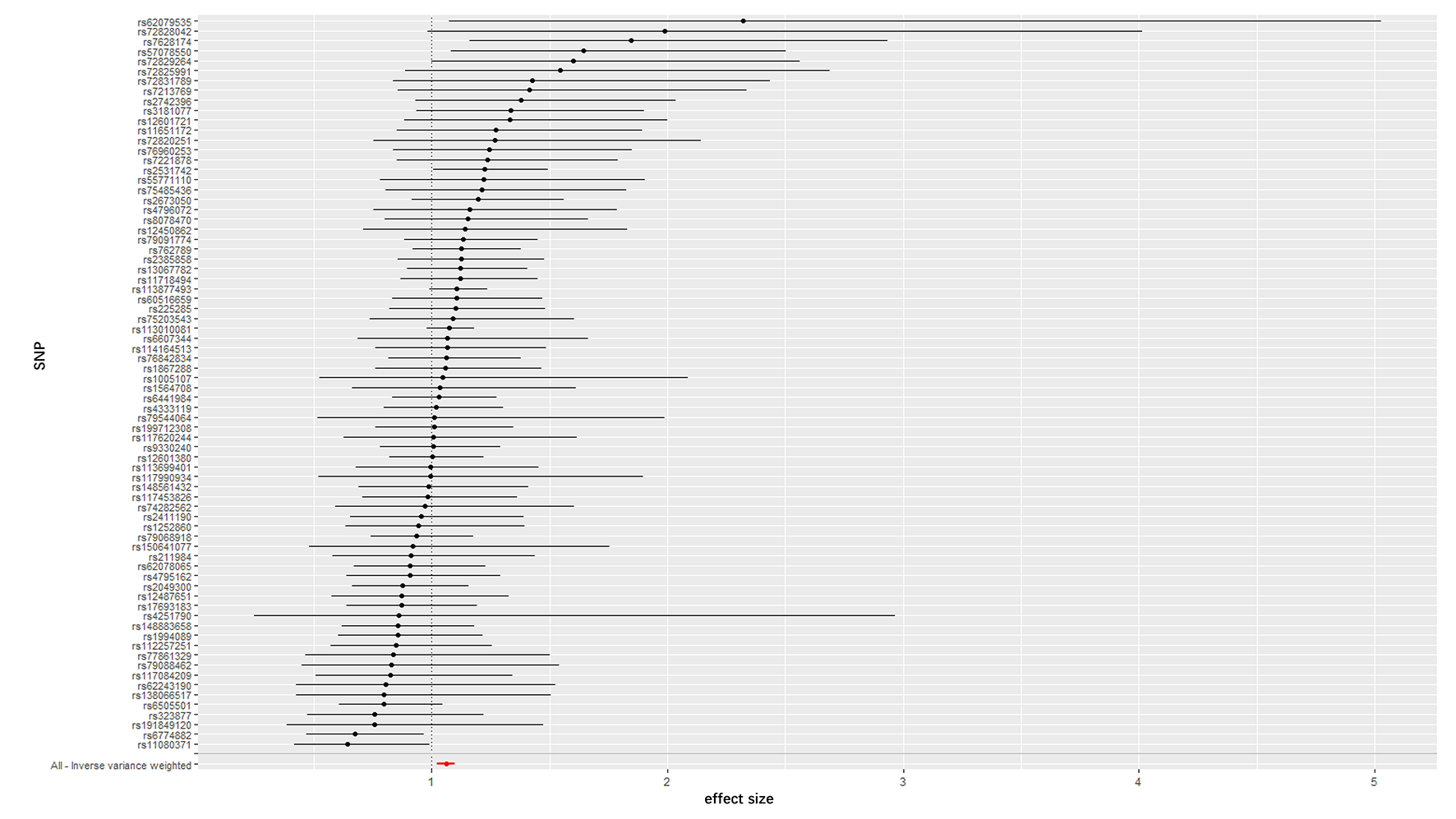

Supplement: Supplementary Figure 1 — Forest plot of the causal effect estimates of MIP1b-associated SNPs on PD risk. Each black dot indicates the causal estimate of MIP1b on PD using an SNP by the Wald ratio method with 95% confidence interval bars. The MR estimate using all SNPs with the IVW method is also shown (red dot with 95% confidence interval bars). PD, Parkinson’s disease; SNP, single-nucleotide polymorphism; MR, Mendelian randomization; IVW, inverse-variance weighted. [file Image_1.TIFF]

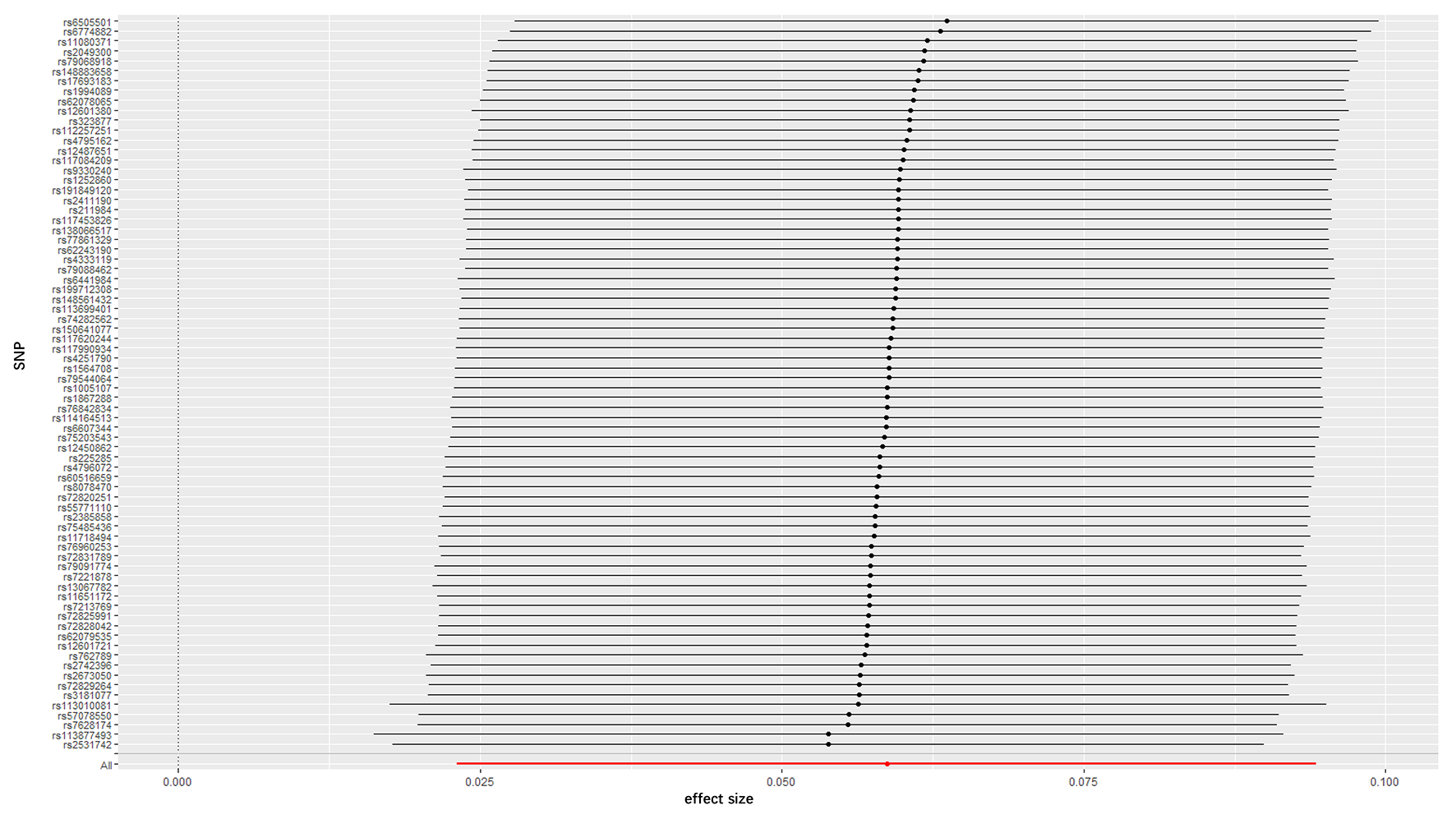

Supplement: Supplementary Figure 2 — Leave-one-out analysis of MIP1b-associated SNPs on PD risk. It was conducted to determine whether the causal effect of circulating MIP1b levels on PD risk was disproportionately influenced by a single SNP. The black dot and bars indicate the MR estimate and 95% confidence interval when the specific SNP is removed. The overall MR analysis, including all SNPs, is also presented for comparison (red dot with 95% confidence interval bars). SNP, single-nucleotide polymorphism; PD, Parkinson’s disease; MR, Mendelian randomization. [file Image_2.TIFF]
