# Supplementary material for: Genetically Predicted Levels of Circulating Inflammatory Cytokines and the Risk and Age at Onset of Parkinson’s Disease: A Two-Sample Mendelian Randomization Study
Source: Front Aging Neurosci. 2022 Mar 1;14:811059. doi: 10.3389/fnagi.2022.811059 (PMC8923644; doi:10.3389/fnagi.2022.811059)
Supplement: Supplementary file 3 [file Data_Sheet_1.DOCX]

Supplementary Material

**Table S1. Summary of detailed information on GWASs and datasets included in our study.**

| Exposures/  Outcomes | Cohorts or datasets | Participants | Publicly available websites |
| --- | --- | --- | --- |
| Inflammatory cytokines | YFS, FINRISK1997, FINRISK2002 | 8,293 Finnish individuals | https://computationalmedicine.fi/data#Cytokine_GWAS |
| PD | IPDGC-NeuroX, UK Biobank, SGPD, IPDGC | 33,674 cases and 449,056 controls of European ancestry | https://bit.ly/2ofzGrk |
| Age at PD onset | IPDGC | 17,996 cases (mostly European adults) | https://drive.google.com/file/d/1n-6eOF6galxP9dLHx_QCndeaOQU2uhCf/view |

PD: Parkinson’s disease; YFS: The Cardiovascular Risk in Young Finns Study; IPDGC: International Parkinson’s Disease Genomics Consortium; SGPD: Systems genomics of Parkinson’s disease consortium.

**Table S2. Detailed information of 19 cytokines and their related SNPs used as instrument variables in the Mendelian randomization study.**

| **Exposure** | **PD risk** | | | **age at onset of PD** | | |
| --- | --- | --- | --- | --- | --- | --- |
|  | **Number of SNPs** | **F statistics median (range)*** | **Variance explained (R^2^)^**^** | **Number of SNPs** | **F statistics median (range)** | **Variance explained (R^2^)** |
| MIP1b | 74 | 47.90 (30.27-789.15) | 0.705 | 65 | 47.46 (30.27-789.15) | 0.630 |
| TRAIL | 25 | 79.13 (32.65-370.01) | 0.329 | 21 | 79.13 (32.65-370.01) | 0.299 |
| IL18 | 8 | 34.32 (31.75-96.17) | 0.105 | 7 | 34.96 (32.06-96.17) | 0.098 |
| MCP1 | 7 | 34.75 (29.97-91.78) | 0.044 | 7 | 34.75 (29.97-91.78) | 0.044 |
| GROa | 6 | 53.64 (30.28-184.38) | 0.131 | 6 | 53.64 (30.28-184.38) | 0.131 |
| Eotaxin | 5 | 39.94 (32.52-95.21) | 0.033 | 4 | 41.99 (34.86-95.21) | 0.027 |
| TNFb | 4 | 45.27 (34.87-79.96) | 0.143 | 2 | 65.44 (50.93-79.96) | 0.104 |
| CTACK | 4 | 51.87 (29.88-142.66) | 0.077 | 4 | 51.87 (29.88-142.66) | 0.077 |
| IL16 | 3 | 40.59 (31.05-131.98) | 0.092 | 3 | 40.59 (31.05-131.98) | 0.092 |
| IL2ra | 3 | 103.12 (39.81-167.61) | 0.151 | 2 | 135.36 (103.12-167.61) | 0.130 |
| IP10 | 2 | 31.58 (31.11-32.04) | 0.019 | 2 | 31.58 (31.11-32.04) | 0.019 |
| IFNg1 | 1 | 32.34 | 0.002 | 1 | 32.34 | 0.002 |
| IL10 | 1 | 37.50 | 0.005 | 1 | 37.50 | 0.005 |
| IL12p70 | 1 | 34.73 | 0.004 | 1 | 34.73 | 0.004 |
| IL17 | 1 | 38.97 | 0.006 | 1 | 38.97 | 0.006 |
| MCSF | 1 | 31.64 | 0.016 | 1 | 31.64 | 0.016 |
| MIF | 1 | 39.05 | 0.011 | 1 | 39.05 | 0.011 |
| MIG | 1 | 42.38 | 0.010 | 1 | 42.38 | 0.010 |
| RANTES | 1 | 29.99 | 0.004 | 1 | 29.99 | 0.004 |

*$F={\beta^{2}}/{{se}^{2}}$, where β is the effect size of the SNPs for inflammatory cytokines and se is the standard error of the SNPs for inflammatory cytokines;

**$R^{2}=\left( \beta\times\sqrt{2\times MAF(1-\mathrm{MAF}}) \right)$2, where MAF is the minor allele frequency and β is the effect size estimates of the SNPs on inflammatory cytokines;

PD: Parkinson’s disease; SNP: single nucleotide polymorphism.
